# The impact of an electronic learning intervention to support appropriate antibiotic prescribing behaviour by non-medical prescribers for upper respiratory tract infections in the primary care setting: a feasibility study

**DOI:** 10.1186/s12913-025-13260-0

**Published:** 2025-08-04

**Authors:** Clare Hawker, Molly Courtenay, Angel Chater, Rose Gallagher, Rosemary Lim, Nicholas Reid, Neil Thomas, Adam DN Williams

**Affiliations:** 1 School of Healthcare Sciences, Cardiff University, Cardiff, UK; 2 Centre for Health, Wellbeing and Behaviour Change, University of Bedfordshire, Luton, UK; 3 University College London Centre for Behaviour Change, London, UK; 4 Royal College of Nursing, London, UK; 5 Reading School of Pharmacy, University of Reading, Reading, UK; 6 Public health Wales, Cardiff, UK; 7 Centre for Trials Research, School of Medicine, Cardiff University, Cardiff, UK

**Keywords:** Antibiotic prescribing, Non-medical prescriber, Upper respiratory tract infections, Theoretical domains framework, COM-B

## Abstract

**Background:**

Interventions are available for general practitioners to support appropriate antibiotic prescribing behaviour for common, acute, uncomplicated, self-limiting upper respiratory tract infections (URTIs). Non-medical prescribers frequently manage these conditions, but no such interventions exist for these groups. This study aimed to assess the feasibility and perceived impact of a theory-based electronic learning intervention designed to support appropriate antibiotic prescribing by non-medical prescribers for URTIs in primary care settings.

**Methods:**

A repeated measures, electronic survey design was used, with data collection occurring pre-intervention (stage 1), post-intervention (stage 3), and at a 3-month follow-up (stage 4). The intervention’s usefulness was assessed by analysing prescribers’ self-reported confidence and knowledge in treating patients with URTIs, as well as their views on the relevance of the intervention to their work. The influence of the intervention on prescribing behaviour was evaluated by examining prescribers’ perceived capabilities, opportunities, and motivations (COM) in the pre-intervention questionnaire and at follow-up. Feasibility outcome measures included recruitment, retention, and engagement with the intervention.

**Results:**

Twenty-one participants (*n* = 18 nurses, *n* = 1 pharmacist, and *n* = 2 paramedics) responded to the recruitment materials, followed the weblink to the survey, and completed the intervention over the 2-month recruitment period. Outcome data for 21 participants were available for pre- and post intervention (stage 1 & 3) and 11 (52%) participants completed follow-up (stage 4). Behavioural practice (information and support for self-management of URTIs) and perceived COM in relation to prescribing for patients with URTIs all increased at follow-up. Mean confidence scores were high at pre- and post-intervention stages but showed a slight decrease at follow-up. The intervention was reported to be useful and applicable to participants’ practice.

**Conclusion:**

It was feasible to recruit the target sample, and participants engaged well with the intervention. However, further consideration is needed for recruiting pharmacists and paramedics and developing a retention strategy for the follow-up survey. Behaviour and COM influences on behaviour changed positively from before to after the intervention. Future work should consider using the intervention in nursing and pharmacy undergraduate programmes, with students on prescribing programmes, and with other non-medical prescribers such as paramedic and physiotherapist prescribers.

**Supplementary Information:**

The online version contains supplementary material available at 10.1186/s12913-025-13260-0.

## Background

Antimicrobial resistance (AMR) is one of the leading causes of mortality worldwide, contributing to 4.95 million deaths and directly causing 1.27 million deaths [1]. Overuse, misuse, and inappropriate disposal of antibiotics in human and animal health accelerates the emergence of AMR in human and animal populations. The United Kingdom (UK) Government’s 20-year-vision for AMR [2], which is aligned to the United Nations Sustainable Development Goals [3], highlights the need for better antimicrobial stewardship (AMS) amongst health professionals, the public, and the pharmaceutical industry. AMS, a multidisciplinary activity, has been defined as “a coherent set of actions which promote the responsible use of antimicrobials” [4], and is linked to a number of behaviours [5, 6].

Common, acute, uncomplicated, self-limiting upper, respiratory tract infections (URTIs) usually resolve spontaneously, with antibiotics in most cases, unlikely to offer clinical benefit [7]. Despite this, most antibiotics are prescribed in primary care for these infections [8, 9] Antibiotic exposure is significantly associated with resistance, and multiple courses of antibiotic treatments are associated with higher resistance rates in patients with URTIs’ [10]. The need to conserve antibiotic efficacy, through the management of URTIs without recourse to antibiotics, is a global priority [10–12], and a key target for interventions is the antibiotic prescribing behaviour of healthcare professionals who manage these infections.

Much research has focused upon trying to understand why general practitioners (GPs) prescribe antibiotics for URTIs. However, GPs are no longer solely responsible for treating and managing URTIs. In the UK, around 50,000 nurses [13], 14,635 pharmacists [14], and around 2000 physiotherapists, podiatrists and paramedics, i.e. allied health professionals (AHPs) [15], have the same independent prescribing capability as doctors. The numbers of these ‘non-medical prescribers’ (NMPs) are steadily increasing [16] to fulfil the workforce needs of the National Health Services (NHS) [17–19]. 

NMPs frequently manage patients with URTIs, with nurses prescribing around 9% of all primary care antibiotics dispensed [20]. It cannot be assumed that the factors influencing GP prescribing in URTI management are the same as those that influence nurse and pharmacist prescribing. For example, nurses tend to have longer consultations and prescribe for less complex patients, while pharmacists may consult with patients outside of a general practice setting. Therefore, it follows that we cannot be certain that interventions targeting the prescribing behavior of GPs will address all the influences on nurse and pharmacist prescribers. Factors such as patient expectations, diagnostic uncertainty, and peer influence play significant roles in their prescribing decisions [21]. To our knowledge, there are currently no interventions specifically designed to support appropriate antibiotic prescribing behavior by these NMPs. This heightens the need to ensure that interventions are also informed by the experiences of nurse and pharmacist prescribers.

We developed a theory-based electronic learning intervention that aimed to support a ‘non- antibiotic prescribing decision’ by NMPs (nurses, pharmacists and allied health professionals (AHPS) for URTIs in primary care. This reflects national prescribing guidance in the UK [7]. Early feasibility work undertaken by our team of researchers identified a need for the addition of complex clinical content (i.e.patient expectations, symptom management, and education to promote self-management) [22]. These additional clinical context were added to our initial intervention. The aim of this study was to assess intervention feasibility and the perceived impact on the prescribing behaviour of NMPs.

## Methods

### Design

A repeated measures, experimental survey design was adopted using electronic surveys.

### Participants

Participants were qualified NMPs, involved in the treatment management of patients with URTIs in a primary care setting in the UK.

### Procedure

Recruitment for the study took place from October to November 2022. RL and NT disseminated information about the study via email through their existing prescriber networks including the Royal College of Nursing General Practice Nursing Forum and the Association for Prescribers, UK. The email included a link to the participant information sheet (PIS) and consent form for those interested in participating. Participants who consented were subsequently sent a link to the survey and intervention. An email address provided within the PIS allowed potential participants to contact the research team with any questions, ensuring they could make an informed decision about participation. Informed consent was obtained electronically using Qualtrics software ©2023, the survey data collection used the same software.

After consenting, participants received a weblink to the online survey which included the pre-intervention questionnaire (stage 1) followed by the e-learning intervention (5 min video embedded within the survey) (stage 2) and the post-intervention questionnaire (stage 3). Participants created an identifier to connect their pre- and post-intervention responses to the follow-up data. Participants were asked to provide their email addresses to be contacted for the follow-up questionnaire (stage 4) which was provided three months after baseline data collection. To maintain confidentiality, email addresses were not linked to questionnaire responses. A weblink was emailed to participants at three months, inviting them to complete the follow-up questionnaire. Participants were unable to re-engage with the intervention during the three-month follow-up period. A sample size calculation was not performed as this was a feasibility study. However, recruitment continued until a maximum of until a maximum of 21 NMPs in total had been recruited; a sample size expected to enable qualitative data saturation [23].

### Intervention

We have developed and tested a theory based electronic learning intervention (an animation) to support and promote appropriate antibiotic prescribing practices among NMPs [22, 23]. The behaviour change wheel (BCW) [24, 25] was used to design the intervention content taking into consideration Capability, Opportunity and Motivation-Behaviour (COM-B) and the Theoretical Domains Framework (TDF) [25, 26]. The COM-B was used to create a behavioural diagnosis that is, what is influencing the behaviour to target for change; and the TDF identified these at a more granular level, based on our previous research [27–29]. The BCW was then used to identify intervention strategies (e.g. education, modelling). In-line with recommendations for interventions that use digital technology [30], an iterative cycle of development and testing was adopted with modifications made to our intervention (specifically the addition of complex clinical content) in the light of questions that arose during earlier testing [22]. The theory-based intervention comprised a 5-minute, interactive, animated scenario of a consultation by a nurse prescriber with a female adult presenting with an URTI. The prescriber depicted in the animation adopted a patient-centred motivational interviewing style [31], to reach a no antibiotic prescribing outcome. To facilitate active learning, NMPs were invited to test their knowledge at the end of the scenario by answering a range of closed questions that focused on the information presented in the scenario.

### Measures

Measures for data collection included demographic details, prescribing behaviour, confidence in practice, application to practice, and feasibility. Table 1 shows data collection points for each measure.

**Table 1:** Data collection at each stage of the study

Measure	Pre-intervention	Post-intervention	Follow-up
Demographic details	X		
Prescribing behaviour	X		X
Confidence in practice	X	X	X
Application to practice		X	

#### Demographic details

Demographic details in the pre-intervention questionnaire included sex, ethnicity, current job title, whether participants were a nurse, pharmacist or AHP, time employed in current position, time qualified as a prescriber, the number of consultations a week in which patients with URTIs were seen, and the percentage for which antibiotics were prescribed.

#### Prescribing behaviour

Perceived COM in relation to the intervention was assessed in the pre-intervention questionnaire and at follow-up. Questions were adapted based on wording formats from Keyworth [32]. These wording formats were designed to fully operationalise all six COM subdomains to evaluate COM-B influences on prescribing behaviour. Participants were initially asked how they would treat a patient who presented with symptoms that suggested an URTI and the key considerations when providing a non-antibiotic alternative for URTIs. Questions were presented as a series of statements and participants were asked to provide a response from 1 (strongly disagree) to 10 (strongly agree) as a measure of behaviour and influences related to COM (See Supplementary Fig. 1). At the end of the statements, participants were invited to add any additional free-text comments related to their responses.

#### Confidence in practice

Further detail on NMPs’ knowledge and confidence managing the treatment of patients presenting with URTIs was assessed in the pre-, post-intervention and follow-up questionnaire using questions developed from our previous work [22]. Participants rated their responses on six items using a 5-point Likert scale from 1 (strongly disagree) to 5 (strongly agree). (See Supplementary Fig. 2).

#### Application to practice

This section was provided only at the post-intervention stage, and comprised of six questions about the usefulness of the intervention and how the information in the intervention may be applied in their working lives. Participants rated their responses on six items using a 5-point Likert scale from 1 (strongly disagree) to 5 (strongly agree) (See Supplementary Fig. 3). Space was provided at the end of each question enabling participants to expand upon their responses. At the end of this section, an open text box asked participants what, if anything, they intended to do with their learning.

#### Feasibility

Feasibility outcome measures included recruitment (and time to reach target sample size), retention (availability of outcome data) and engagement with the intervention.

### Data analysis

Descriptive statistics (frequencies and percentages) were used to characterise participants’ prescribing behaviour for patients presenting with URTIs (range, median, mean, Standard Deviation (SD)), confidence in prescribing antibiotics (range, median, mean, SD) and their views on the practical application of the intervention (median, mean, SD). Change scores were calculated between each data collection point. Quantitative data were analysed using SPSS V.25 [33]. Content analysis [34] was used to analyse free-text comments, and explore qualitative findings. This process involved initial identification of commonly occurring themes, representing the range of responses. Themes were then broken down into mutually exclusive and exhaustive categories, and responses were assigned to categories and coded. The frequency of different responses was then counted. This process was performed manually by MC.

## Results

A total of 21 participants responded to the study invitation, consented, and completed study stages 1 to 3 (pre-intervention questionnaire, e-intervention, and post-intervention questionnaire, 11 (52%) were retained at stage 4 (follow-up questionnaire). Table 2 presents NMPs’ demographic data.

**Table 2:** Non-Medical prescribers’ demographic details

	Stages 1–3 Pre/Post intervention (*n* = 21)		Stage 4 Follow-up (*n* = 11)	
	Frequency	%	Frequency	%
Gender				
Man	2	9.5	.	.
Woman	19	90.5	11	100.0
Ethnicity				
White British	19	90.5	10	90.9
Asian/Asian British	1	4.8	.	.
Another ethnic group	1	4.8	1	9.1
Type of non-medical prescriber				
Nurse	18	85.7	100	100.0
Pharmacist	1	4.8	.	.
Other (Paramedics)	2	9.5	.	.
Time in post (years)				
< 1	1	4.8	.	.
1–4	10	47.6	5	45.5
5–10	5	23.8	3	27.3
> 10	5	23.8	3	27.3
Time as qualified prescriber (years)				
< 1	1	4.8	.	.
1–4	7	33.3	2	18.2
5–10	5	23.8	3	27.3
> 10	8	38.1	6	54.5
Number of URTI consultations URTIs per week				
< 1	1	4.8	.	.
1 to 5	4	19.0	.	.
6 to 10	4	19.0	.	.
11 to 20	7	33.3	.	.
> 21	5	23.8	.	.
Antibiotics prescribed to presenting URTIs (percent)				
0	3	14.3	.	.
1–10	6	28.6	.	.
11–20	5	23.8	.	.
21–30	0	0.0	.	.
31–40	3	14.3	.	.
41–50	4	19.0	.	.

Of those who completed all study stages, all were nurses, and 6 (55%) had been qualified as a prescriber for over 10 years. In the pre-intervention questionnaire, 12 (57%) reported having over 10 URTI consultations per week. Three (14%) participants reported never prescribing antibiotics in these consultations whereas 5 (23.8%) reported prescribing antibiotics for between 11 and 20% of their consultations.

### Prescribing behaviour

Free text comments to the question ‘How would you treat a patient who presented with symptoms that suggested a URTI?’ included: (1) examination of the patient (including a review of their medical history) and assessment of risk factors and whether the infection is bacterial or viral (*n* = 8); (2) the provision of advice and information (including what to do if symptoms continue/worsen) along with an explanation for a non-antibiotic prescribing decision and answering any questions the patient may have (*n* = 8); and (3) self-management advice including analgesia, rest, and fluids. This advice was determined by patient co-morbidities, and whether monitoring/testing was required (*n* = 15).

Key factors participants reported they would consider when providing a non-antibiotic alternative for URTIs included: (1) advice on self-management (analgesia, rest, fluids, throat spray/lozenges and saline throat gargle) (*n* = 7); (2) patient characteristics i.e. age, comorbidities, frailty and results from examination along with the patient’s social situation (*n* = 16).

Table 3 describes participants’ responses to prescribing behaviour statements at pre-intervention and follow-up. There were high levels of agreement with all statements at the pre-intervention stage. Statement 3 (physical capability) received the highest agreement with a mean score of 9.1. Statements 1 (behaviour), 2 (psychological capability), 6 (reflective motivation) and 7 (automatic motivation) each received high agreement with scores between 8.5 and 8.9. Statements 4 (physical opportunity) and 5 (social opportunity) had the lowest agreement with mean scores of 7.7 and 7.9 respectively. There was an increase in the levels of agreement across all statements in the follow-up questionnaire. The highest increase in these levels across the two surveys were for statements: 7 (automatic motivation), 4 (physical opportunity), 5 (social opportunity), and 6 (reflective motivation). Six participants provided additional comments related to their prescribing behaviour. Time was identified as important when making a non-antibiotic prescribing decision (*n* = 4). This time was required to explain decisions, provide self-management advice, and inform patients what to do if symptoms did not dissipate or worsened. This was exacerbated in elderly patients, if the decision was required to be explained to a carer/family member, or if there was a language barrier.

*“Time is the critical factor which impacts on the consultation. It can also pose a challenge in terms of printing information leaflets. Another factor is language barrier - translation is often via telephone which removes the social cues. It would be advantageous if there were patient information leaflets in different languages.”* (Participant 1).

One participant admitted giving into the will of ‘adamant patients’ and prescribing antibiotics. By contrast, another stated that they would only prescribe antibiotics if the case required antibiotics i.e. unless the CENTOR score (National Institute for Health and Care Excellence (NICE) 2018) (a score which predicts which patients will have culture confirmed streptococcal infections of their pharynx) is triggered.

**Table 3:** Prescribing behaviour and influences related to capability, opportunity and motivation in the management of URTIs

Prescribing Behaviour Statements	Stage 1 Pre intervention (*n *= 21)			Stage 4 Follow-up (*n *= 11)			Mean change
	Range	Median	Mean (SD)	Range	Median	Mean (SD)	
1) I provide information and support for self-management for URTIs in all relevant consultations.	1, 10	10	8.6 (2.4)	5, 10	10	9.1 (1.6)	0.5
2) I am psychologically able to provide information and support for self-management of URTIs in all relevant consultations.	4, 10	9	8.9 (1.5)	8, 10	9	9.4 (0.7)	0.5
3) I am physically able to provide information and support for self-management of URTIs in all relevant consultations.	4, 10	10	9.1 (1.5)	8, 10	10	9.5 (0.7)	0.4
4) I have the physical opportunity to provide information and support for self-management of URTIs in all relevant consultations.	5, 10	8	7.7 (2.0)	4, 10	9	8.5 (2.0)	0.8
5) I have the social opportunity to provide information and support for self-management of URTIs in all relevant consultations.	5, 10	8	7.9 (1.8)	4, 10	9	8.9 (1.8)	1.0
6) I am motivated to provide information and support for self-management of URTIs in all relevant consultations.	5, 10	10	8.7 (1.7)	8, 10	10	9.6 (0.7)	0.9
7) Providing information and support for self-management of URTIs is something I do routinely in all relevant consultations.	4, 10	9	8.5 (1.8)	8, 10	10	9.6 (0.7)	0.9

### Impact on prescribing confidence

Table 4 presents participants’ confidence scores in managing patients presenting with URTIs at pre-intervention (stage 1), post-intervention (stage 3), and follow-up (stage 4). Neutral (3 = neither agree nor disagree) to very high levels of confidence were reported for all statements at both pre- and post-intervention, with mean confidence for each statement being high. The follow-up data has a wider range in confidence from very low to very high. At the post intervention point there was slight increases in mean confidence levels for statements 1 (patient expectations), 2 (supporting patients understand), 5 (skills to help patients see different viewpoints) and 6 (patients happy with prescribing decisions). There was no change for statement 3 (building rapport) and 4 (communication skills) (which were high at 4.6 and 4.7 respectively). At follow-up mean confidence scores were still high but there were slight decreases in confidence for all statements apart from statement 3 (building rapport) which stayed the same.

**Table 4:** Confidence in the treatment of URTIs

Confidence questions	Stage 1: Pre intervention (*n* = 21)			Stage 3: Post intervention (*n* = 21)			Mean change	Range	Stage 4: Follow-up (*n* = 11)		Mean change*
I am confident that I…	Range	Median	Mean (SD)	Range	Median	Mean (SD)			Median	Mean (SD)	
1)…can gain information from these patients on their expectations.	3, 5	5	4.5 (0.6)	3, 5	5	4.6 (0.6)	0.1	2, 5	4	4.3 (0.9)	−0.2
2)…can support these patients to comprehend the health information that I give them.	3, 5	4	4.4 (0.6)	3, 5	5	4.5 (0.6)	0.1	1, 5	5	4.4 (1.2)	0
3)…can build rapport with these patients.	3, 5	5	4.6 (0.6)	3, 5	5	4.6 (0.6)	0.1	1, 5	5	4.6 (1.2)	0
4)…have the skills needed to communicate effectively with patients with URTIs.	3, 5	5	4.7 (0.6)	3, 5	5	4.7 (0.6)	0	1, 5	5	4.6 (1.2)	−0.1
5)…have the skills to get these patients to see and examine different viewpoints.	3, 5	4	4.3 (0.6)	3, 5	5	4.5 (0.6)	0.2	1, 5	5	4.3 (1.2)	0
6)…patients both understand and are happy with my prescribing decision.	3, 5	4	4.0 (0.6)	3, 5	4	4.4 (0.6)	0.4	1, 5	5	4.3 (1.2)	0.3

*mean change from pre-intervention

Of the prescribers who provided an open text response, the most common factor that impacted the confidence of the prescriber in making their decision, was patient expectations for an antibiotic (*n* = 6), culture (i.e. it was normal in their country of origin to buy antibiotics over-the-counter and so there was the feeling of being deprived of normal treatment) (*n* = 1), greater prescribing experience (i.e. more experience provided more confidence to make a non-antibiotic prescribing decision) (*n* = 2).

### Application to practice

Prescribers agreed that the information presented in the intervention was applicable to their practice, was useful to them as a prescriber and encouraged them to consider how they would apply the information in their practice. Prescribers disagreed that the information in the intervention was new to them, although responses ranged from strongly disagree to strongly agree. Prescribers were neutral to statement 6 (the information presented encouraged me to think differently) (see Table 5).

**Table 5:** Usefulness and application of the intervention to practice

Application to practice	Stage 4: Post intervention (*n* = 21)		
In my opinion, the information presented…	Range	Median	Mean (SD)
1) …was mostly new to me.	1, 5	1	1.7 (1.0)
2) …was applicable to my practice.	2, 5	5	4.3 (1.1)
3) …was useful to me as a prescriber.	3, 5	5	4.4 (0.7)
4) …has made me feel more comfortable speaking with patients with RTIs.	1, 5	4	3.9 (0.9)
5) …has encouraged me to consider how I would apply the information in my practice.	2, 5	3	4.0 (0.9)
6) …has encouraged me to think differently.	1, 5	3	2.9 (1.1)

When asked what participants intended to do with their learning, responses included: (1) share with team members/colleagues (*n* = 5); (2) use it to reinforce their current practice (*n* = 3); and (3) use the information in future consultations (*n* = 1).

### Feasibility

Feasibility items were assessed against findings with implications indicated for a potential future research to assess effectiveness of the intervention. With regards to feasibility outcomes, it was feasible to recruit the target sample. However, the recruitment of pharmacists was low, and future work should give further consideration to the recruitment strategy for this group. Outcome data was collected for the target sample for study stages 1–3, but retaining participants at follow-up was more challenging. Further consideration needs to be given to a retention strategy for this stage of the study. All participants engaged with the intervention, this high engagement and intention to share information with colleagues highlights its acceptability and potential for broader application. Details in Table 6.

**Table 6:** Feasibility assessment

Fidelity item	Finding	Implications of findings for future research
Recruitment	21 NMPs (18 nurses, 1 pharmacist, 2 paramedics) recruited over 2 months	Further consideration needs to be given to the recruitment strategy, specifically targeting the recruitment of pharmacists.
Retention	All those who consented completed stages 1 to 3. Only 52% completed stage 4 (follow-up questionnaire).	It is feasible to collect data across each of the four study stages. However, further consideration is needed for a retention strategy for study stage four.
Engagement with intervention	All participants engaged with the intervention video and completed the post intervention measures.	The high engagement with the intervention and intention to share information with colleagues highlights its acceptability and potential for broader application.

## Discussion

The recruitment of 21 NMPs (18 nurses, 1 pharmacist, and 2 paramedics) within a two-month period demonstrates the feasibility of engaging healthcare professionals in studies aimed at improving antibiotic prescribing practices. However, the low numbers of pharmacists and paramedics highlights a need for targeted recruitment strategies to ensure a more balanced participation across different prescriber groups. Future studies should consider tailored approaches to engage various groups of NMPs. The retention rate was only 52% for the follow-up questionnaire. This attrition rate is a common challenge in studies and underscores the importance of developing robust retention strategies.

The demographic data revealed that the majority of participants were experienced nurses, with over half having more than 10 years of prescribing experience. This level of experience likely contributed to the high baseline confidence and knowledge reported by participants. The variability in prescribing practices, with some participants never prescribing antibiotics for URTIs and others prescribing them in up to 20% of consultations, indicates a need for interventions that address both ends of the prescribing spectrum. Tailoring interventions to different levels of experience and prescribing habits could enhance their effectiveness.

The qualitative data on prescribing behavior provided valuable insights into the decision-making processes of NMPs. The emphasis on patient examination, risk assessment, and self-management advice aligns with best practices in antimicrobial stewardship. Similar to previous findings [35], undertaking a physical examination, patient centred management strategies (including advice and support for self-management and what to do if symptoms get worse) were strategies employed when used within a consultation when a non-antibiotic prescribing decision was made. Behaviour (e.g. providing information) and the COM influences to behaviour (physical opportunity, social opportunity, reflective and automatic motivation) changed from before to after the intervention. Time (physical opportunity) and patient expectations (social opportunity) were identified from qualitative data as important influences on prescribing behaviour. The influence of patient characteristics and social situations on prescribing decisions highlights the complexity of clinical practice. Interventions should incorporate training on these contextual factors to support prescribers in making informed, patient-centered decisions.

The study found high levels of confidence among participants in managing URTIs, with slight increases post-intervention. The intervention is only one 5-minute clinical scenario, and not repeated. The follow-up data showed a wider range of confidence levels, suggesting that while the intervention had an immediate positive impact, maintaining this confidence over time may require ongoing support. The factors impacting confidence, such as patient expectations and cultural norms, point to the need for continuous education and resources to help prescribers navigate these challenge. Future work should involve designing resources (including patient information leaflets and posters, and educational material) specifically to support intervention content and to be made available for use alongside the intervention. This will both inform the patient and also encourage the prescriber that patients will not be seeking antibiotics.

Participants reported that the intervention was applicable and useful for their practice, with intentions to share the information with colleagues and apply it in future consultations. However, participants were unable to access the intervention once they had completed it and reported that it would have been helpful for them to do so. Additionl access may have helped to sustain confidence levels during the 3 month follow-up period and something that should be considered in future work. NMPs’ agreed that the information presented in the intervention was applicable to practice and useful, however despite adding additional clinical content, as in our earlier work [22], participants disagreed that the information was new to them. This could be because the majority of respondents had more than 5 years experience as a qualified NMP. The information would be less likely to be familiar to those who are training to becomeNMPs. Furthermore, Standards for the Initial Education and Training Standards (IETS) of Pharmacists in the UK [36] will see student pharmacists become prescribers at the point of registration from summer 2026 onwards and nurses should be ‘prescriber ready’ upon registration [37, 38]. As well as the use of our intervention in prescribing programmes, future work should also consider the use of our intervention in undergraduate pharmacy and nursing programmes.

Patients expectations for an antibiotic and greater prescribing experience were each factors reported to impact the confidence of the prescriber with regards to a non-antibiotic prescribing decision. Given that 43% of patients expect to receive an antibiotic from nurse and pharmacist prescribers [36], and that nurses are now able to access prescribing training with as little as one years qualified experience [38] and pharmacists will become prescribers at the point of registration from summer 2026 [36], this highlights the importance of interventions specifically to support appropriate antibiotic prescribing behaviour by these groups.

### Strengths and limitations

The recruitment of pharmacist and paramedic prescribers was challenging. Pharmacist and paramedic prescribers may not have considered themselves to fit the inclusion criteria. For example, we know that pharmacists tend to run clinics that are specific to a particular chronic condition, and that there are fewer pharmacist prescribers than nurse prescribers, with fewer community dispensed antibiotics written by pharmacists [16]. Further consideration to pharmacist and paramedic sample size will therefore be needed to inform future research. Our sample was an opportunistic sample and therefore, possibly more biased to appropriate prescribing. Actual prescribing behaviour was not able to be measured thus effectiveness of the intervention could not be commented on.

## Conclusion

It was feasible to recruit the target sample and particpants engaged with the intervention, however further consideration needs to be given to the recruitment of pharmacists and paramedic prescribers, and a retention strategy for the follow-up survey. Behaviour (provide information and support for self-management for URTIs) and the COM influences to behaviour (physical opportunity, social opportunity, reflective and automatic motivation) changed from before to after the intervention. However low numbers mean that it is difficult to identify if this change is meaningful. Future work should assess the effectiveness of this intervention in a randomised control trial.

## Supplementary Information

Supplementary Material 1.

Supplementary Material 2.

## Data Availability

All data generated or analysed during this study are included in this published article.

## Abbreviations

AHPs: Allied Health Professions
AMR: Antimicrobial resistance
AMS: Antimicrobial stewardship
BCW: Behaviour change wheel
COM: Capabilities, opportunities, and motivations
COM-B: Capability, Opportunity and Motivation-Behaviour
GPs: General practitioners
NMPs: National Health Services (NHS).National Institute for Health and Care Excellence (NICE)Non-medical prescribers’
PIS: Participant information sheet
SD: Standard Deviation
TDF: Theoretical Domains Framework
UK: United Kingdom
URTIs: Upper, respiratory tract infections
