# Supplementary material for: The impact of an electronic learning intervention to support appropriate antibiotic prescribing behaviour by non-medical prescribers for upper respiratory tract infections in the primary care setting: a feasibility study
Source: BMC Health Serv Res. 2025 Aug 4;25:1022. doi: 10.1186/s12913-025-13260-0 (PMC12323022; doi:10.1186/s12913-025-13260-0)
Supplement: Supplementary file 1 — Supplementary Material 1. [file 12913_2025_13260_MOESM1_ESM.docx]

Supplementary Figure 1 – Prescribing behaviour survey questions

- I provide information and support for self-management for URTIs in all relevant consultations (behaviour)
- I am psychologically able (i.e I have the knowledge and/or psychological skills, strength or stamina to engage in the necessary thought processes to engage in the activity concerned) to provide information and support for self-management of URTIs in all relevant consultations (psychological capability)
- I am physically able (i.e. I have the physical skill, strength, or stamina to engage in the activity concerned) to provide information and support for self-management of URTIs in all relevant consultations (physical capability)
- I have the physical opportunity (ie. I have sufficient time, sufficient space, the necessary materials and resources, reminders) to provide information and support for self-management of URTIs in all relevant consultations (physical opportunity)
- I have the social opportunity (i.e. Interpersonal influences, social cues and cultural norms) to provide information and support for self-management of URTIs in all relevant consultations (social opportunity)
- I am motivated to provide information and support (i.e. I intend to, I have made a plan to, I am confident I can, I have the desire to, I feel the need to, it is part of my role and identity to) for self-management of URTIs in all relevant consultations (reflective motivation)
- Providing information and support for self-management of URTIs is something I do routinely (i.e. it is something I do before I realise I’m doing it, it is part of my routine) in all relevant consultations (automatic motivation)

Supplementary Figure 2 - Confidence survey questions

- Gain information on patient expectations
- Support patients
- Build rapport
- Communicate effectively
- See and examine different viewpoints
- Ensure that patients both understand and are happy with the prescribing decision

Supplementary Figure 3 – Application to practice survey questions

- Whether the information was known to participants
- Its applicability to practice
- If the intervention would be useful to them as prescribers
- Whether it makes them feel more comfortable when speaking with patients with RTIs
- If it encourages participants to consider how they would apply the information to practice
- If it encourages participants to think differently
